# Preimplantation genetic testing for aneuploidy (PGT-A) vs. morphology-based blastocyst selection for transfer: cost-effectiveness analysis within Israel’s publicly funded IVF system

**DOI:** 10.1186/s13584-026-00790-z

**Published:** 2026-11-02

**Authors:** Einat Zivi, Moshe Leshno, Ariel Hammerman, Tomer Ziv-Baran

**Affiliations:** 1 School of Public Health, Gray Faculty of Medical and Health Sciences, Tel-Aviv University, Tel-Aviv, Israel; 2 Coller School of Management, Tel-Aviv University, Tel-Aviv, Israel; 3 Department of Epidemiology and Preventive Medicine, School of Public Health, Gray Faculty of Medical and Health Sciences, Tel- Aviv University, Tel-Aviv, Israel

**Keywords:** Preimplantation genetic testing for aneuploidy, Cost-effectiveness, Incremental cost, Blastocyst

## Abstract

**Background:**

Israel operates one of the most extensive publicly funded in vitro fertilization (IVF) programs worldwide. Alongside IVF, adjunct technologies aimed at improving reproductive outcomes, such as preimplantation genetic testing for aneuploidy (PGT-A), have been increasingly adopted. PGT-A is designed to identify chromosomally normal embryos before transfer. Despite its growing use, PGT-A is not included in Israel’s National Health Basket. Given the local high utilization of IVF and the substantial public expenditure, evaluating the economic value of PGT-A is essential for evidence-based health policy decision-making. Most available analyses of PGT-A originate from private or mixed healthcare systems, where treatment costs and reimbursement structures differ from Israel’s fully publicly funded IVF model. This study aimed to determine whether PGT-A is cost-effective when provided as part of Israel’s IVF program.

**Methods:**

A theoretical model-based cost-effectiveness analysis using a Markov decision framework compared PGT-A with morphology-based embryo selection from the perspective of the Israeli public healthcare payer. The model simulated repeated embryo transfers for various age groups and blastocyst availability scenarios. Relevant cost estimates of the two strategies were based on Israeli Ministry of Health data (2025). The probabilities of main clinical outcomes were derived from the American Society for Assisted Reproductive Technology (SART) database. The effectiveness outcome was live birth. Deterministic one-way sensitivity analyses were performed.

**Results:**

The cost-effectiveness analysis showed dominance for the no-PGT-A strategy; PGT-A was associated with higher costs and lower effectiveness compared with no-PGT-A. Across all age groups and blastocyst numbers, PGT-A was consistently more costly than no-PGT-A. Sensitivity analyses identified the cost of the PGT-A test per tested embryo and the number of available blastocysts as the most influential factors on the incremental cost. It was demonstrated that although PGT-A is a less effective strategy, reducing the costs of the PGT-A test could make it a less costly strategy for women aged 38 years and above. In this circumstance, a trade-off between strategies can be considered.

**Conclusions:**

PGT-A cannot be recommended for public funding within the Israeli public IVF program. Its application may be considered for advanced-age patients if the cost of the PGT-A test is reduced.

## Background

In vitro fertilization (IVF) success is influenced by various factors, with one key determinant being the embryo’s chromosomal integrity, particularly the presence or absence of aneuploidy. Aneuploid embryos, which carry an abnormal number of chromosomes, are the leading cause of implantation failure, miscarriages, and failed IVF cycles. The likelihood of aneuploidy increases with maternal age, making age one of the strongest predictors of IVF outcomes. The rate of aneuploidies rises steeply after age 37 and exceeds 70% by the 40s. Likewise, IVF success rates decline sharply with maternal age, and the likelihood of an embryonic arrest or implantation failure rises [1].

Traditionally, IVF embryologists select embryos for transfer according to morphological grading criteria. This assessment method is based on visual characteristics, including cell numbers, symmetry, and fragmentation [2]. While morphological assessment has been widely used, it is limited in its ability to accurately predict an embryo’s ploidy status [3–7]. Due to the substantial impact of the embryo ploidy status on IVF outcomes, preimplantation genetic testing for aneuploidy (PGT-A) has been developed as an adjunct technique to IVF, aiming to screen embryos for chromosomal abnormalities. PGT-A involves culturing embryos to the blastocyst stage (day 5/6 after fertilization), followed by a biopsy of several trophectoderm cells and DNA extraction, which is then analyzed using advanced genetic techniques. PGT-A has emerged as a commonly used technology within contemporary reproductive medicine, aiming to prioritize embryos by identifying euploid blastocysts for transfer. Nonetheless, the effectiveness of PGT-A remains a subject of debate in clinical practice. Evidence suggests that PGT-A may reduce miscarriage rates; however, its impact on live birth rates has not been conclusively proven [8–11].

From an economic evaluation perspective, the cost-effectiveness of PGT-A is influenced by multiple factors, including the perspective analyzed (e.g., patients vs. payers), demographics, healthcare system structures, and costs. Several international studies have evaluated the cost-effectiveness of PGT-A in specific clinical settings and population groups using theoretical and empirical models, which have yielded heterogeneous results. Theoretical analytical models have reported that PGT-A is cost-effective for women of advanced maternal age who can develop multiple blastocysts [12–13]. Similarly, a “real-life” study by Neal et al. [14] demonstrated that PGT-A could be cost-effective for women over 38 years old, due to reduced miscarriage rates and time to pregnancy. Likewise, Lee et al. [15] reported that PGT-A was cost-effective for women with recurrent IVF failures, although its benefits were less pronounced in younger women with a good prognosis. Additionally, other studies have found that although PGT-A may be clinically advantageous, it is rather expensive and not cost-effective for most women undergoing IVF [16–17]. These findings suggest that while PGT-A may not be universally cost-effective or suitable for routine application, it may be of value for selected patient groups, particularly advanced age women with sufficient embryos available for testing.

The economic burden of IVF varies substantially across healthcare systems and according to the perspective considered [18]. In the United States (U.S.), where IVF has historically been largely financed out-of-pocket, reported prices to patients for a single IVF cycle range from approximately $10,000 to $25,000, and multiple cycles are often needed, resulting in a total per-live-birth expenditure that can exceed $60,000 [19]. These figures primarily represent prices faced by patients rather than estimates of the underlying resource costs of providing IVF. Insurance coverage varies considerably across states, and IVF utilization has been reported to be higher in states with comprehensive insurance mandates than in those without such mandates [19].

Among publicly funded healthcare systems, ART utilization, coverage, eligibility criteria, and associated economic burden vary considerably. In France, 118,394 ART treatment cycles were reported in 2019, corresponding to approximately 10 cycles per 1,000 women aged 15–45 years, while in Belgium, 34,440 ART cycles were reported in the same year, corresponding to approximately 16.1 cycles per 1,000 women aged 15–45 years [20]. Both countries provide public funding for ART, although access and reimbursement are subject to defined age-related restrictions. In France, public funding is available up to age 42 for oocyte retrieval and up to age 44 for embryo transfer, whereas in Belgium public funding is limited to women aged ≤ 45 years; Belgian legislation permits oocyte retrieval up to age 45 and embryo transfer up to age 47 [21]. In France, the broader economic burden of infertility treatment has been estimated at approximately €7,000 per treated woman over 3.5 years, corresponding to €70 million per 10,000 women [22]. 

Thus, even among publicly funded healthcare systems, ART utilization, eligibility criteria, and the extent of public coverage vary substantially. Israel presents a distinctive case, possessing one of the highest per-capita rates of IVF globally. According to the Israeli National IVF Registry [23], Israel maintains one of the highest rates of IVF treatment worldwide. In 2021, approximately 51,000 IVF cycles were registered, which is around 30 (27–34) cycles per 1,000 women at reproductive age - substantially exceeding the utilization rates reported in other publicly funded healthcare systems such as France and Belgium, as well as the U.S. rate of 6.7 cycles per 1,000 women at reproductive age [20, 24]. Furthermore, in Israel, women undergoing IVF are generally older; approximately 30% of IVF cycles are performed on women above 40 years, compared with 20% in the U.S. [23–24]. Consequently, a substantial proportion of the IVF cycles in Israel are performed on women of advanced maternal age, who face greater fertility challenges due to higher aneuploidy rates and reduced ovarian reserve. As a result, achieving a live birth often requires multiple IVF cycles.

The IVF volume of procedures in Israel is placing a significant burden on the local healthcare resources. Given that artificial reproductive technologies (ART) treatments are publicly funded through Israel’s national healthcare system, evaluating the cost-effectiveness of PGT-A from the perspective of the Israeli healthcare payer is markedly important.

Under Israel’s National Health Insurance, IVF is covered for women up to age 45 attempting to conceive their 1st and 2nd child. Although the number of publicly funded IVF cycles in Israel is generally not strictly limited, continuation of treatment is subject to specific criteria following repeated cycles without achieving embryo transfer. The overall cost for the public payers of an IVF cycle can exceed 20,000 New Israeli Shekels (NIS) based on the Ministry of Health (MoH) tariff [25]. This includes coverage for ovarian stimulation follow-up visits, oocyte retrieval procedure, micromanipulation at the IVF lab, and, if relevant, frozen embryo transfer costs. Fertility drugs are also covered with a low patient co-payment. The cost of fertility medication for one cycle can reach thousands of NIS, depending on the patient’s age and/or ovarian response. Elective add-ons such as PGT-A are typically excluded from the Health Basket and require private payment. In contrast to Israel, in countries with predominantly private healthcare systems (e.g. the United States), PGT-A has become more widely adopted. The broader insurance and reimbursement structures in such settings often make PGT-A more accessible, with relatively low price per embryo tested. Although PGT-A is not routinely integrated into IVF treatment in Israel and remains an elective add-on procedure, multiple IVF cycles and embryo transfers are publicly funded, making the economic context different.

While several studies have evaluated the economic aspects of PGT-A, analyses specific to Israel are lacking. This study aims to address this gap by conducting a cost-effectiveness analysis of PGT-A within the Israeli healthcare system context. Given the global increase in PGT-A utilization and the ongoing debate about its value in ART, this study aims to provide actionable insights for clinicians, policymakers, and patients, taking into account the unique demographic profiles and policy factors that contribute to a significant public expenditure on IVF in Israel.

## Methods

**Fig. 1:**
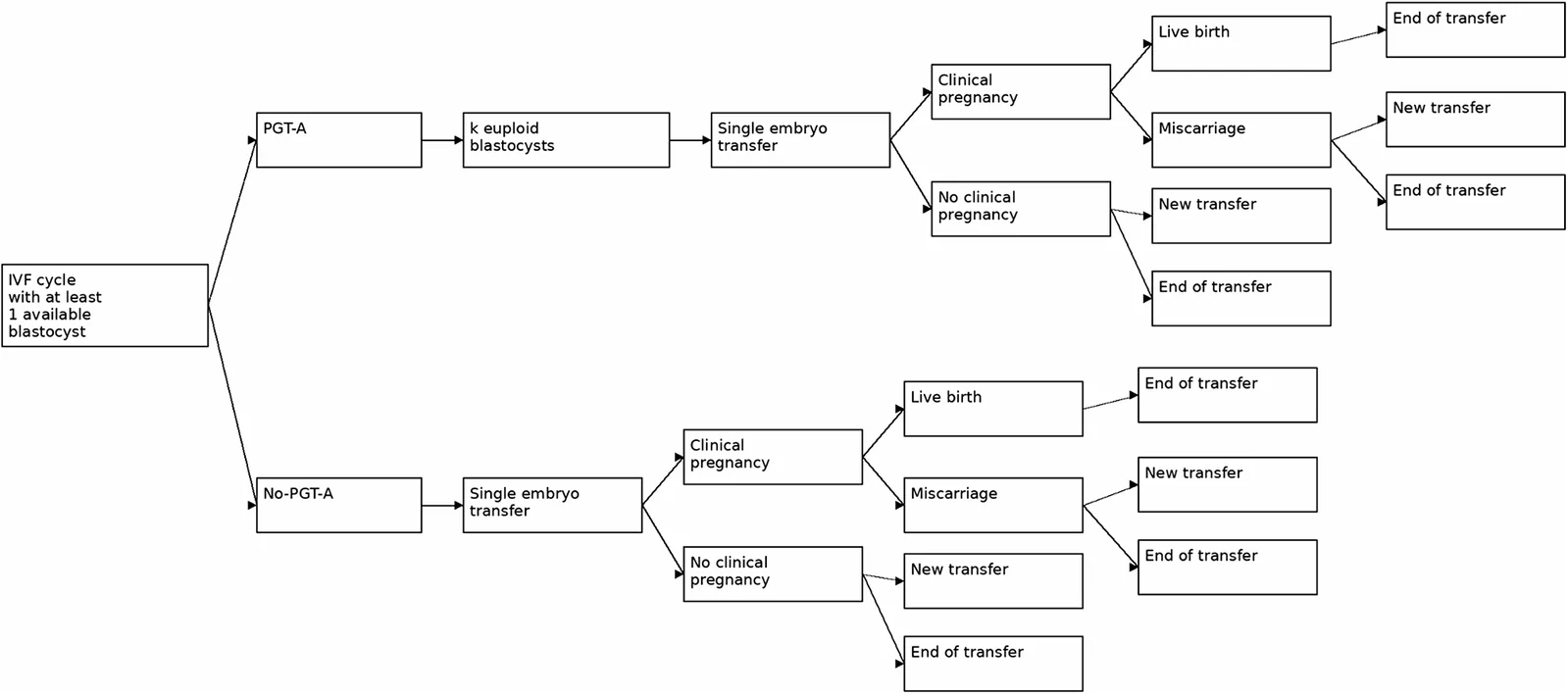
The cost-effectiveness Markovian model. Women were entered into the model based on the initial number of usable blastocysts available (ranging from 1 to 8) for biopsy or transfer without PGT-A. At each cycle, transitions between health states were determined by age-specific probabilities. This process continued until either a live birth occurred or all available blastocysts had been used. The model also accounted for the probabilities of having one or more euploid blastocysts following PGT-A (k-number of euploid blastocysts)

The study employed a Markovian model to assess the cost-effectiveness of public funding for PGT-A in Israel. The model was developed to compare two treatment strategies: (1) Single embryo transfer (SET) after PGT-A, which included embryo biopsy on day 5/6, cryopreservation, and sequential SET of thawed euploid blastocysts derived from the initial biopsied cohort, and (2) SET of all available blastocysts without PGT-A. The Markovian model simulates the clinical pathway as transitions between health states (continue transferring blastocysts or end of transfer), to allow realistic modeling of repeated transfer attempts (Fig. 1). The model was developed and analyzed using TreeAge Pro (Version 2026, TreeAge Software, LLC, Williamstown, MA, USA), with additional calculations and analyses performed using MATLAB (MathWorks, Natick, MA, USA).

Women undergoing IVF entered the model according to the given initial number of usable blastocysts (ranging from 1 to 8) and transitioned between health states according to age-specific probabilities at each model cycle. The model incorporated three key health conditions - clinical pregnancy (defined as the presence of a intrauterine gestational sac detected by ultrasound), miscarriage (defined as pregnancy loss after intrauterine pregnancy was confirmed by ultrasound), and a live birth. In instances where a clinical pregnancy was not achieved, or a miscarriage occurred (hence, resulting in no live birth), an additional frozen embryo transfer was performed contingent upon the availability of blastocysts. The endpoints of the Markovian model were defined as either the achievement of a live birth or the exhaustion of all available blastocysts.

In the PGT-A strategy, all first transfers were frozen. In the no-PGT-A strategy, the first transfer could be either fresh or frozen. In both strategies, the model accounted for additional frozen SET attempts derived from the same blastocysts’ initial batch in cases where no clinical pregnancy was achieved or a miscarriage occurred. The model’s time horizon was defined as the period from the point at which the number of available blastocysts is determined until the first live birth occurs or the original blastocyst cohort is exhausted.

The cost assessment was conducted from the healthcare provider’s perspective in Israel (the National Health Basket) and included direct medical costs based on the MoH tariff. The costs included in the model according to the MoH tariff were the prices of embryo thawing and transfer, and the prices associated with medical or surgical miscarriage (presented in Table 1). In this context, MoH tariff reflect regulated procedure prices rather than actual resource costs. The cost of the diagnostic PGT-A test per tested embryo was estimated from prices obtained from several Israeli fertility clinics. The first decision node of the model was blastocyst availability. Accordingly, any costs incurred before blastocysts were available were considered irrelevant and excluded from the model. These costs include hormonal treatments for ovarian stimulation, oocyte retrieval procedure, and in vitro fertilization. Nevertheless, these costs are assumed to be similar between strategies. As the analysis was performed from the healthcare payer’s perspective, costs from the patient’s perspective, such as work absence and follow-up visits, were not included. Due to the relatively short time horizon, costs were not discounted. Costs are presented in NIS and are referenced to 2025.

The study population comprised women undergoing transfer of blastocysts derived from autologous oocytes. The model evaluated different age groups within the study population. For each age group, probability estimates were derived from relevant published literature. Since no inclusive local data exist in Israel that differentiate between types of embryo transfer (fresh versus frozen) or regarding PGT-A outcomes, the transition probabilities for the model were sourced from published literature and presented in Table 1. The probabilities included key parameters such as the likelihood of obtaining an euploid embryo in an IVF-PGT-A cycle using Next Generation Sequencing (NGS) for testing [26], while considering the patient’s age and the number of blastocysts available for biopsy. The model considered the probabilities for additional euploid blastocyst transfers in cases where the previous transfer resulted in no clinical pregnancy or a miscarriage. The probabilities for clinical pregnancy, miscarriage, and live birth associated with the two treatment strategies were based on data derived from the Society for Assisted Reproductive Technology Clinic Outcome Reporting System (SART-CORS) database [27, 28]. The probabilities for women up to 40 years were based on a recent large cohort study that included outcomes of the first cycles in the SART-CORS from 2016 to 2019 [27] and on published clinical outcomes of the SART from 2021 to 2023 [28, 29].

The no-PGT-A strategy considered the overall probability that the first transfer would be either fresh (without the associated freezing and thawing costs) or frozen. There are several clinical scenarios in which a fresh embryo transfer is not performed. These include, for example, suboptimal endometrial receptivity (e.g., inadequate endometrial thickness), risk of ovarian hyperstimulation syndrome (OHSS), presence of intrauterine fluid, elevated progesterone levels on the day of trigger, or other maternal health considerations that necessitate delaying transfer and opting for a freeze-all strategy. The fresh-versus-frozen transfer probabilities were sourced from the Gertner Institute’s registry in Israel report [23] and the SART database. According to the Israel report, about 60% of transfers are fresh, and 40% are frozen. Data from the SART database also indicate comparable proportions in various age groups after the first ovum pick up (OPU). For reasons of simplicity and given the absence of significant differences, the clinical outcome probabilities (clinical pregnancy and miscarriage rates) for the first embryo transfer without PGT-A were derived by averaging the published probabilities for both fresh and frozen embryo transfers. In this regard, it is worth noting that there is literature, including a Cochrane review [30], indicating comparable clinical outcomes between fresh and frozen transfers. Live birth rates were calculated per established clinical pregnancy that did not result in a miscarriage (rather than per transfer).

**Table 1:** Costs in NIS and Probabilities included in the model by age groups

Costs and Probabilities	Description	< 35	35–37	38–40	41–42
PGT-A costs	Cost of PGT-A per blastocyst tested	2,500	2,500	2,500	2,500
Frozen embryo transfer costs	Cost of frozen embryo transfer according to the Ministry of Health prices list for 2025	5,500	5,500	5,500	5,500
Miscarriage management costs	Cost of miscarriage management according to the Ministry of Health prices list for 2025, assuming an 80 − 20 ratio between medical and surgical management	2,850	2,850	2,850	2,850
Clinical pregnancy - PGT-A	Probability of clinical pregnancy following an euploid blastocyst transfer	0.6	0.6	0.6	0.6
Clinical pregnancy - no-PGT-A	Probability of clinical pregnancy following no-PGT-A transfer	0.58	0.53	0.45	0.32
Miscarriage - PGT-A	Probability of miscarriage after clinical pregnancy confirmed following an euploid blastocyst transfer	0.14	0.14	0.14	0.14
Miscarriage -no-PGT-A	Probability of miscarriage after clinical pregnancy confirmed following no-PGT-A transfer	0.15	0.2	0.26	0.45
Live birth - PGT-A	Probability of a live birth per euploid clinical pregnancy that did not result in a miscarriage	0.86	0.86	0.86	0.86
Live birth - no-PGT-A	Probability of a live birth per non-PGT-A clinical pregnancy that did not result in a miscarriage	0.85	0.8	0.74	0.55
Euploidy rates	Probability of an euploid embryo	0.6	0.5	0.38	0.27

Several central assumptions were incorporated into the model. For the PGT-A arm, it was assumed that no aneuploid or mosaic (two or more cell populations with different chromosomal complements) embryos would be transferred, that biopsy results would be conclusive (rather than “no results”), that no additional sampling would be required, and that embryo biopsy would not adversely affect implantation rates. For both the PGT-A and no-PGT-A strategies, it was assumed that all transfers involved a single embryo and that the biopsied and/or transferred embryos would have good morphological grading and be defined as usable blastocysts [31]. The model assumes that around 80% of early miscarriages will be successfully treated medically and 20% by surgical evacuation [32–34]. General model assumptions included complete survival of frozen embryos after thawing and the exclusion of rare events, such as ectopic pregnancy and intrauterine fetal death. To simplify the model, pregnancy outcomes following an established clinical pregnancy were not further stratified according to prenatal diagnostic findings (chorionic villus sampling or amniocentesis). Specifically, the model did not include a separate pathway for pregnancy termination following an abnormal prenatal finding. Also, the model assumed that all subsequent transfers would have clinical outcome probabilities comparable to those of the initial transfer. Consequently, we avoided the uncommon clinical scenario of repeated implantation failure (RIF), which represents less than 5% of the IVF population [35].

The model first assessed the incremental cost and incremental effectiveness per live birth for each strategy, stratified by age group and number of available blastocysts. To evaluate model robustness, a series of sensitivity analyses was conducted for each age group. First, a Tornado diagram was used to explore uncertainty and to determine which model parameters had the most impact on the incremental cost and in what way. The diagram ranks key model parameters according to their impact on the incremental cost between PGT-A and morphology-based embryo selection. The parameters considered were the quantity of available blastocysts, associated costs, probabilities of main clinical outcomes, and the likelihood of a fresh first transfer in the no-PGT-A approach (p_fresh). Additional sensitivity analyses evaluated the effect of key influential parameters on the incremental cost and incremental cost-effectiveness ratio (ICER).

## Results

The model demonstrated that PGT-A is more costly than no-PGT-A, and the probability of a live birth with PGT-A is lower than with no-PGT-A. Therefore, the no-PGT-A dominates the PGT-A strategy (lower cost and higher probability of live birth), irrespective of the patient’s age group or the number of available blastocysts.

**Fig. 2:**
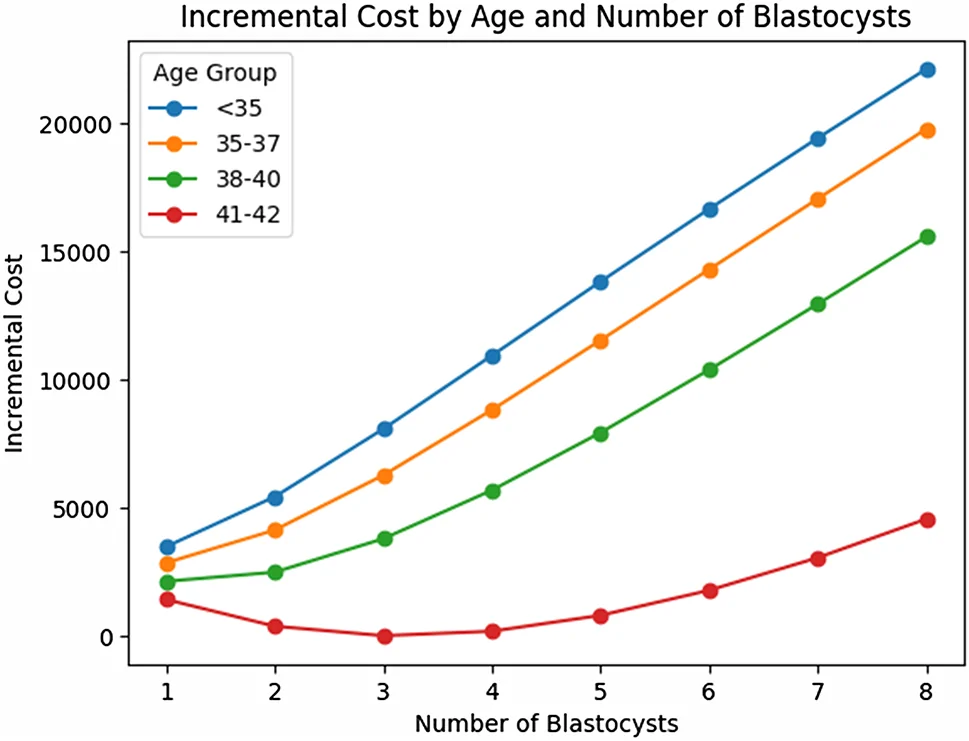
Incremental cost between strategies by blastocyst number and age groups . For each blastocyst count, the graph depicts the incremental cost in NIS per live birth

**Fig. 3:**
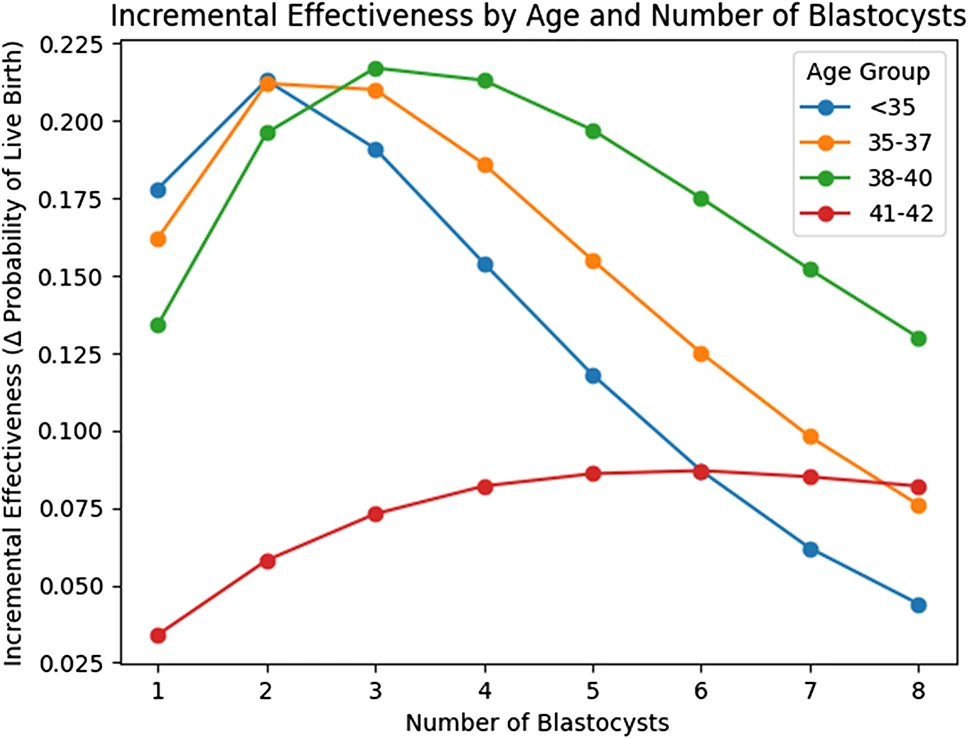
Incremental probabilities of live birth between strategies by blastocyst number and age groups. Incremental probability values show that in all scenarios, PGT-A is less effective than the no-PGT-A strategy

Incremental cost values showed that, in all scenarios, PGT-A is more costly (Fig. 2). Additionally, the probability of a live birth with PGT-A was always found to be less than or approximately equal to that without PGT-A (Fig. 3). Therefore, we focused on the cost outcomes of these strategies, aiming to evaluate under which scenarios PGT-A may be economically viable.

**Fig. 4:**
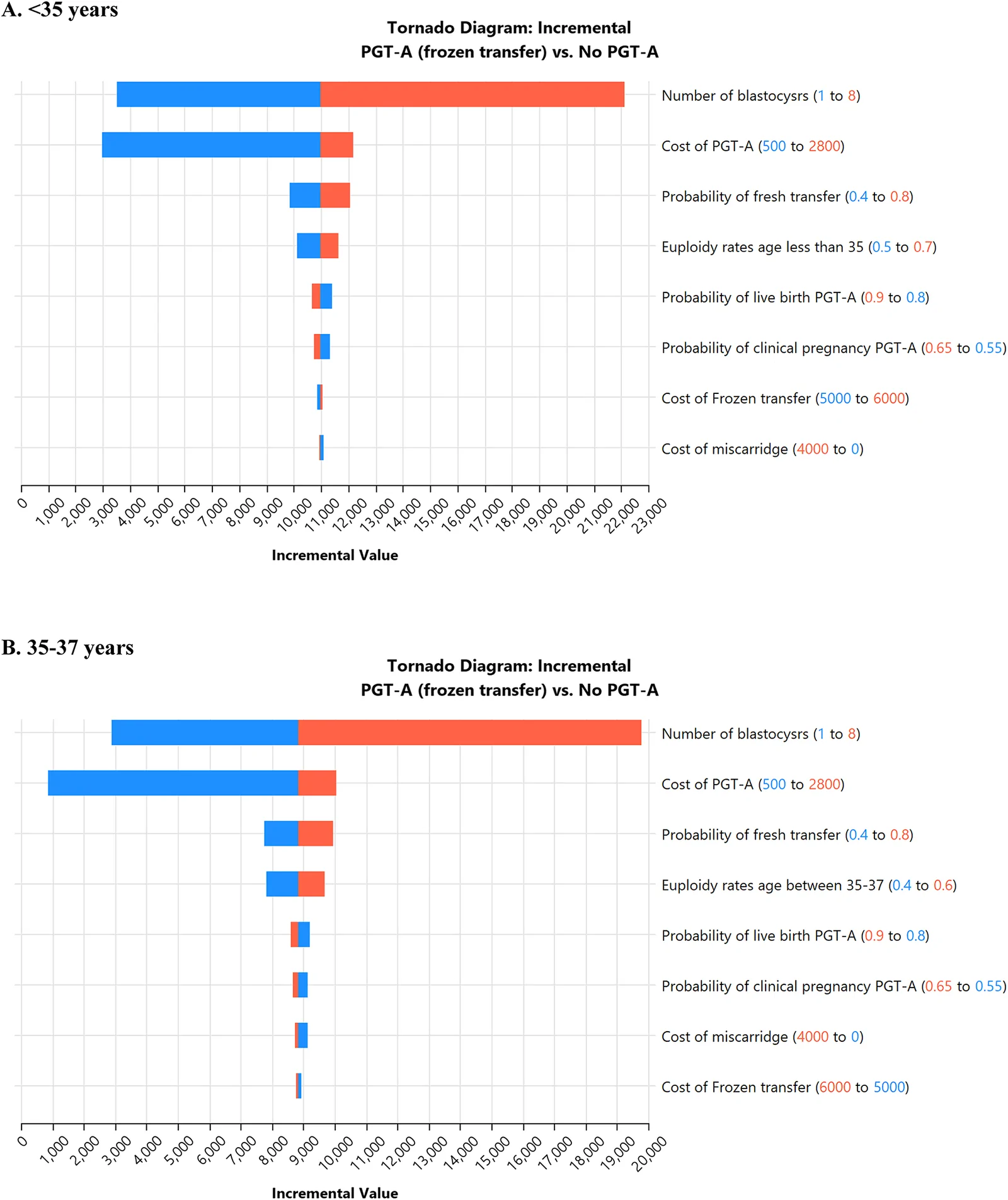

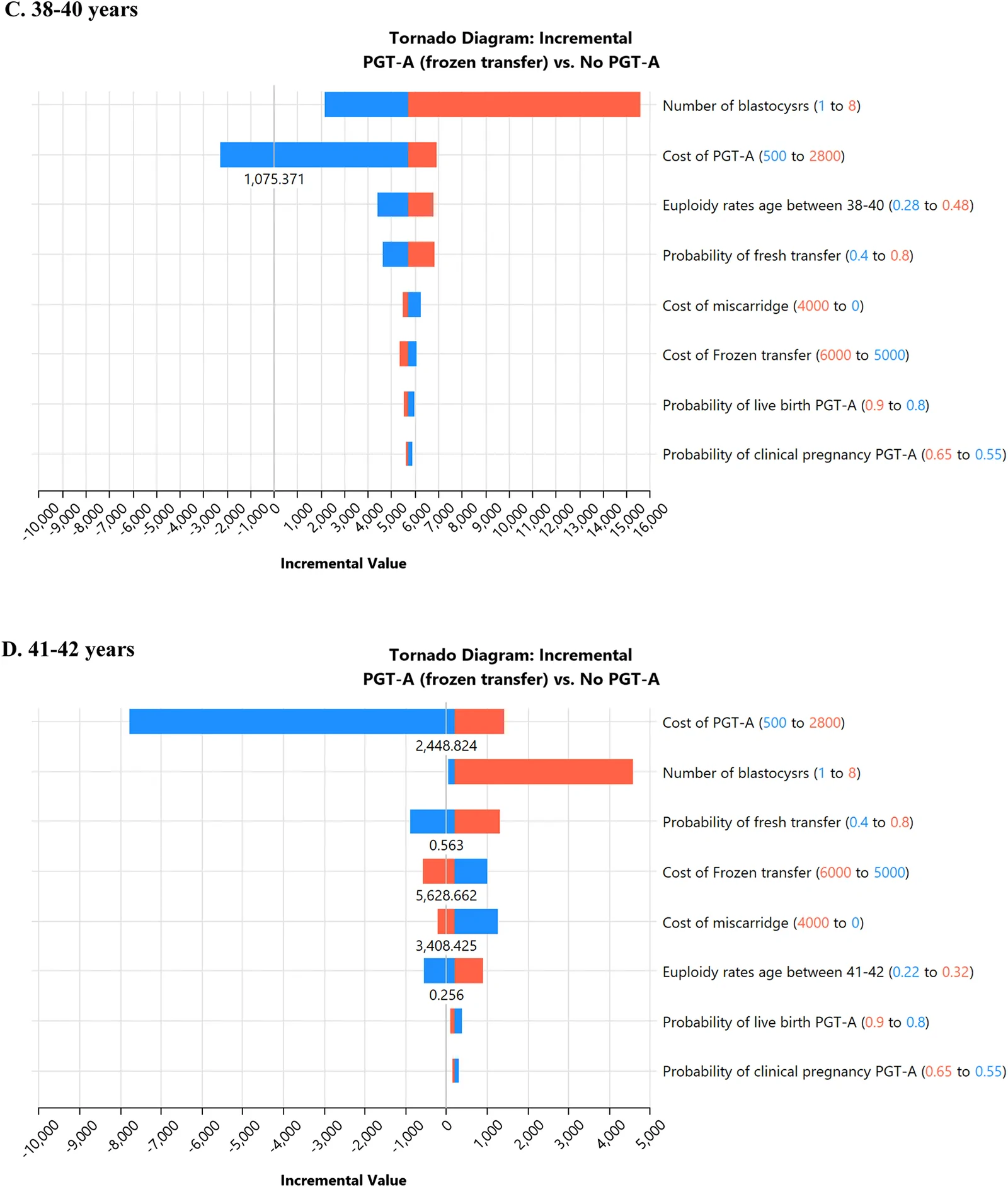
Tornado diagrams by age group for women with four available blastocysts. The diagram ranks key model parameters according to their impact on the incremental cost between PGT-A and morphology-based embryo selection. The vertical line represents the expected incremental cost value. Each bar represents the effect of varying one parameter within its plausible range on the incremental cost. Red bars indicate that increasing the parameter raises the incremental cost, while blue bars show the opposite trend. The length of each bar reflects the magnitude of influence; longer bars correspond to parameters that contribute most to uncertainty in the model’s economic outcome

A series of sensitivity analyses was subsequently performed to assess the model’s robustness. First, a Tornado diagram (Fig. 4) was conducted for each age group at the event, where four blastocysts were available (as a base case condition). The Tornado diagrams demonstrated that the incremental cost for women under 40 years was most sensitive to the number of available blastocysts, followed by the cost of the PGT-A test per tested embryo (Fig. 4A–4C).

**Fig. 5:**
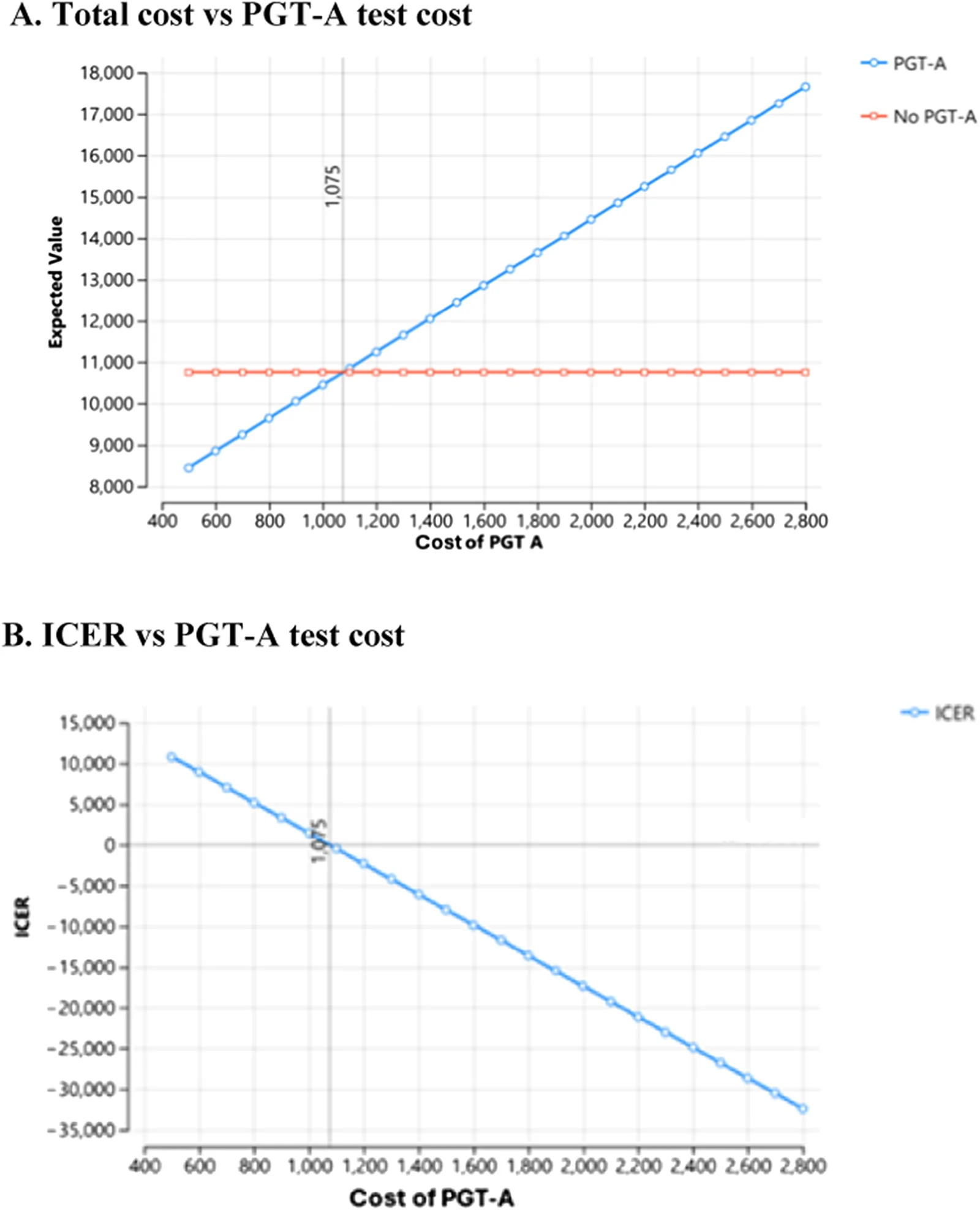
One-way sensitivity analyses for women aged 38–40 years with four blastocysts available. Panel** A** depicts the expected total cost value as a function of the PGT-A test cost, comparing PGT-A (blue) with no-PGT-A (red). The Y-axis represents the total cost of each strategy, while the X-axis represents the cost of the PGT-A test. The vertical distance between the blue and red lines reflects the incremental cost between strategies. The intersection point is at 1,075 NIS. Below this price, PGT-A is less costly; above it, it becomes more expensive. Panel** B** depicts the ICER value as a function of the PGT-A test cost per embryo tested. The ICER becomes zero at a PGT-A cost of 1,075 NIS (incremental cost = 0). Below this price, PGT-A remains less effective but less costly (positive ratio). Above this price, PGT-A is more costly and less effective (negative ratio)

For women under 37 years, the Tornado diagram indicated that the PGT-A strategy provides no savings. However, for the 38-40-year age group, should the cost of the PGT-A test be reduced to 1,075 NIS per tested embryo, the overall costs associated with each strategy would become comparable (Fig. 4C). Subsequently, a one-way sensitivity analysis confirmed that below 1,075 NIS, the PGT-A strategy was less costly than no PGT-A (Fig. 5A). An additional one-way sensitivity analysis showed that below 1,075 NIS, the ICER values became positive, indicating that PGT-A was less costly but still less effective than the no-PGT-A strategy (Fig. 5B).

**Fig. 6:**
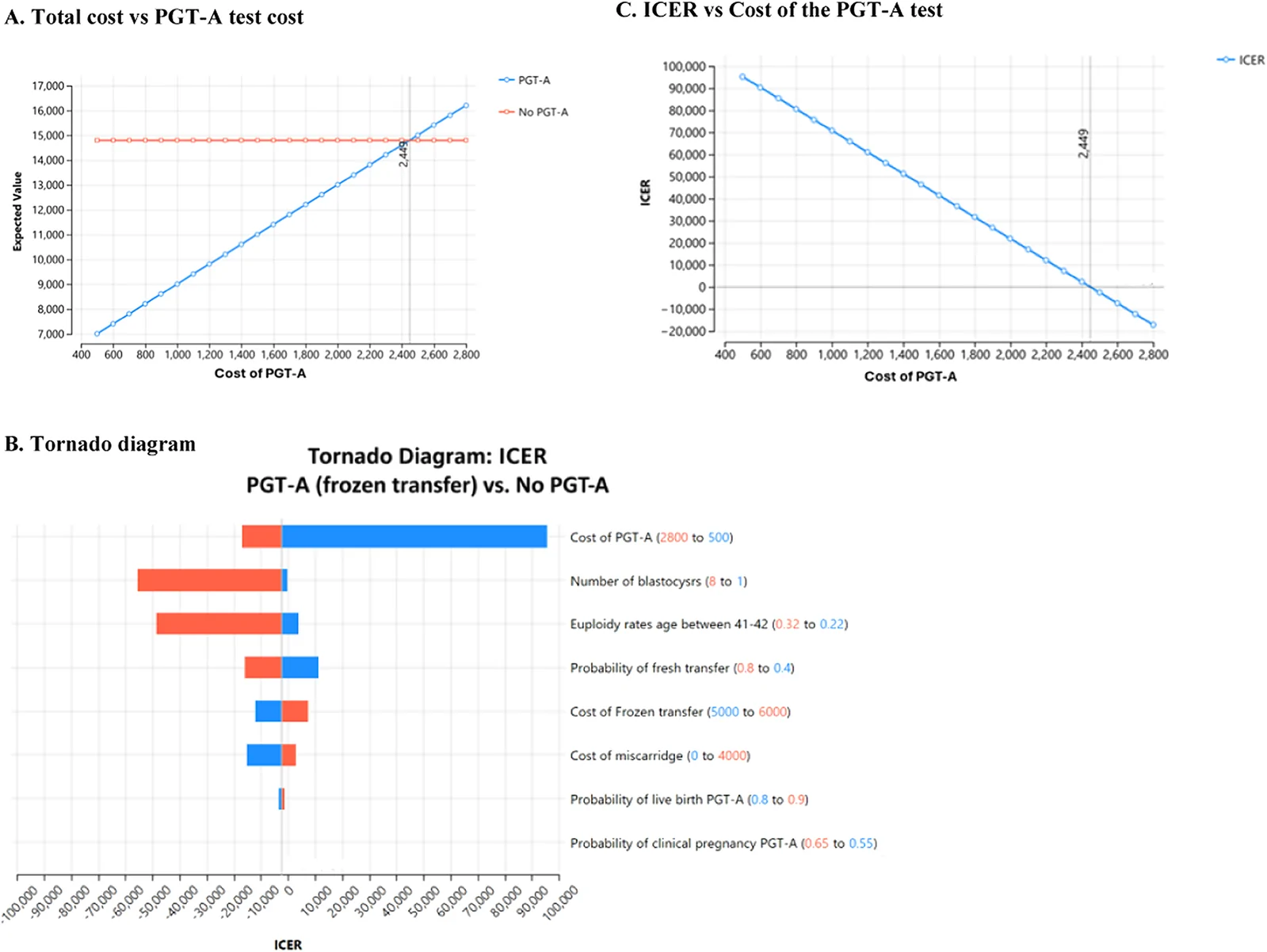
One-way sensitivity analyses for women aged 41–42 years with four blastocysts available. Panel** A** depicts the expected total costs value as a function of the PGT-A test cost per embryo tested, comparing PGT-A (blue) with no-PGT-A (red). The Y-axis represents the total cost of each strategy, while the X-axis represents the cost of the PGT-A test. The vertical distance between the blue and red lines reflects the incremental cost between strategies. The intersection point is at 2,449 NIS. Below this price, PGT-A is less costly; above it, it becomes more expensive. Panel** B** presents a Tornado diagram that ranks key model parameters according to their impact on the ICER of PGT-A versus morphology-based embryo selection. Each bar represents the effect of varying one parameter within its plausible range on ICER. Red bars indicate that increasing the parameter raises the ICER, while blue bars show the opposite trend. The length of each bar reflects the magnitude of influence - longer bars correspond to parameters that contribute most to uncertainty in the model’s economic outcome. Panel** C** depicts the ICER value as a function of the PGT-A test cost. The ICER becomes zero at a PGT-A cost of 2,449 NIS (incremental cost = 0). Below this price, PGT-A remains less effective but less costly (positive ratio). Above this price, PGT-A is more costly and less effective (negative ratio)

For the 41-42-year age group, the Tornado diagram indicated that the cost of PGT-A was the most influential factor on the incremental cost, followed by the number of available blastocysts (Fig. 4D). Comparable total costs between strategies would be achieved if the PGT-A test cost decreased to approximately 2,449 NIS per embryo tested, which is close to the current cost in several Israeli IVF clinics. One-way sensitivity analysis confirmed that below a test cost of 2,449 NIS per embryo, the PGT-A strategy was less costly than the no-PGT-A strategy (Fig. 6A). An additional tornado diagram validated that the cost of the PGT-A test is the most influential factor on ICER (Fig. 6B). Furthermore, one-way sensitivity analysis showed that above a test cost of 2,449 NIS per embryo, the ICER was negative, indicating that PGT-A was more costly and less effective. Below 2,449 NIS, PGT-A was less costly but still less effective than the no-PGT-A strategy (Fig. 6C).

Overall, the analyses indicated that for women over 38 years old, the PGT-A strategy could become less costly if the PGT-A test cost is reduced, with even minimal reductions observed for women aged 41–42. However, the effectiveness of PGT-A was consistently inferior to that of the no-PGT-A approach.

## Discussion

This study provides an assessment of the economic value of PGT-A through a decision-analytic cost-effectiveness model specifically designed for the Israeli healthcare system.

The study demonstrated that, under base-case parameters, the ‘no-PGT-A’ strategy was cost-saving across all age groups, offering higher effectiveness at a lower total cost per live birth. Accordingly, the ‘no-PGT-A’ was found to be the dominant strategy under current conditions. However, the economic gap between strategies narrows as maternal age advances. The model also identified the price of the PGT-A test per tested embryo and the number of available blastocysts as the primary determinants of the incremental cost and ICER. Subsequently, the study suggests that PGT-A may become less costly (though still less effective) if the cost of PGT-A testing decreases for women of advanced age. In this scenario, PGT-A would shift from being dominated on the cost-effectiveness plane to the trade-off quadrant. In this circumstance, PGT-A may offer, at best, a reduction in total costs, but it does not demonstrate an advantage in live birth rates.

In Israel, PGT-A is not considered a standard practice in IVF units, and a substantial reduction in testing costs is unlikely in the near future. In contrast to Israel, in the U.S., PGT-A is routinely integrated into IVF cycles and is considered the standard of care in several clinics [36]. As a result, the cost per tested embryo is relatively low, making the procedure economically viable; while in Israel, the price is approximately four times higher. Nevertheless, sensitivity analyses indicate that for women in the 41–42 age group, even a slight reduction in the current test cost could shift PGT-A to the “trade-off” zone. Whether such a trade-off is acceptable depends on the health system’s priorities and its willingness to tolerate a marginal loss in effectiveness for potential cost advantages.

While PGT-A may not increase clinical success rates, its selective use could still be justified if it contributes to other outcomes, such as a shorter time-to-pregnancy, fewer miscarriages, or a reduced emotional and logistical burden on patients. From a policy perspective, a targeted, prognosis-based funding approach—rather than routine, universal application across all women undergoing IVF —may represent a balanced compromise between economic efficiency and patient-centered care. Such a strategy reflects a pragmatic, value-driven approach to incorporating PGT-A into publicly financed IVF programs.

This analysis was motivated by the need to reduce avoidable costs and treatment burden. Our results operationalize this rationale: by lowering the cost of PGT-A and providing it for cases with a higher pretest probability of aneuploidy (primarily in individuals over 38 years), payers may achieve better value while minimizing unnecessary procedures. International practice patterns align with our findings: payers and professional societies, such as ASRM in the U.S. and the Human Fertilization and Embryology Authority (HFEA) in England, are encouraging the selective, prognosis-based use of PGT-A-particularly in older women, rather than its universal application [11, 37].

An important distinction in interpreting PGT-A effectiveness and, consequently, its cost-effectiveness lies in the evaluation metric. When measuring clinical outcomes per embryo transferred, PGT-A significantly improves clinical pregnancy and live birth rates by transferring only euploid blastocysts. However, this measurement does not consider the low euploidy rates observed in women of advanced age. Thus, the distinction between per transfer to per blastocyst availability is conceptually important. The strength of our analytical model lies in its consideration of the probability of having one or more euploid blastocysts for transfer, based on both patient age and the availability of blastocysts for biopsy. The study was modeled based on blastocyst availability pre-biopsy, and the results indicated that PGT-A did not demonstrate cost-effectiveness. However, if analysis had been conducted based on the availability of post-biopsy blastocysts (i.e., one transfer cycle of one euploid blastocyst vs. one transfer cycle of a morphologically selected blastocyst), PGT-A would have appeared to be cost-effective for the overall IVF population. This would inaccurately simplify the complexities involved in performing PGT-A and overstate its clinical benefits. Nonetheless, in clinical settings where embryo selection for transfer is necessary, such as healthcare systems that limit the number of funded IVF cycles or embryo transfers, PGT-A may confer a relative advantage, particularly when outcomes are evaluated on a per-transfer basis. Under these constraints, prioritizing euploid embryo transfers can improve efficiency and reduce the number of non-viable transfers. However, this rationale does not apply to the Israeli context, where patients are generally permitted unlimited embryo transfers. In such a system, the clinical utility of PGT-A is markedly reduced, as all embryos can ultimately be transferred without imposed restrictions.

Although this study contributes a new economic perspective regarding the cost- effectiveness of PGT-A in Israel, several limitations must be acknowledged. The model did not include early IVF events, such as ovarian response and fertilization rate, that substantially affect blastocyst availability and the success rate per oocyte retrieval. Women with low ovarian response, especially at advanced age, may require multiple IVF cycles and oocyte retrievals to achieve usable blastocysts. Accordingly, earlier costs associated with IVF treatment (e.g., ovarian stimulation and OPU) are not represented in the model. Although one IVF cycle may be sufficient to reach transfer without PGT-A, achieving an euploid embryo, particularly for poor prognosis women, may require several cycles, each of which incurs significant expenses. Additionally, the model suggests that the decision to perform PGT-A occurs after blastocyst formation, whereas in clinical practice, it is made much earlier, even before the cycle begins. Moreover, physicians may advise women with poor ovarian response to transfer embryos at earlier developmental stages (i.e., cleavage stage), avoiding extended culture in the lab to the blastocyst stage and the risk of transfer cancellation, and/or advise avoiding SET and transferring more than one unscreened embryo to increase chances for success. These limitations underscore the need for a more comprehensive and nuanced analytical model to evaluate the cost-effectiveness of PGT-A compared with no-PGT-A per oocyte retrieval, while accounting for ovarian response and embryonic development outcomes.

Furthermore, our model is limited in its data resources of parameters. The cost of the PGT-A test for the healthcare provider (i.e. MoH) was evaluated according to prices obtained from several major Israeli fertility clinics. However, MoH tariffs represent regulated prices rather than actual resource costs. The price of the PGT-A test may change following its inclusion in the health basket through price regulation, negotiation with providers or suppliers, or risk-sharing agreements. Nonetheless, our model included sensitivity analyses across a broad PGT-A price range, including prices substantially lower than the current Israeli price. Thus, the analysis explicitly examined how reductions in the PGT-A price affect the economic comparison, rather than assuming that the current price is fixed. Moreover, while costs reflect the local Israeli healthcare system, clinical outcomes are based on SART data, which may not represent Israeli demographics and clinical outcomes. Nevertheless, possible discrepancies were taken into consideration in the Tornado diagrams performed. Also, the SART database is retrospective and may overrepresent women with favorable prognoses. Patients with RIF or other challenging conditions, such as recurrent pregnancy losses, are underrepresented in this cohort, nor in global literature. Consequently, these subgroups could not be incorporated into the model, and it cannot be ruled out that analyzing other populations with subfertility might have yielded different conclusions. In this context, women older than 42 years are also underrepresented in the SART database. This is particularly relevant to the Israeli setting, where publicly funded IVF is available up to age 45, whereas our analysis was restricted to women aged ≤ 42 years. Accordingly, the findings may not be generalizable to women aged 43–45 years, who constitute a clinically relevant subgroup within the Israeli publicly funded IVF system. In addition, the age-specific euploidy rates incorporated into the model were derived from a single published source and may not fully reflect variability across laboratories, PGT-A platforms, or populations. As these probabilities determine the proportion of embryos considered eligible for transfer in the PGT-A arm, variation in these estimates could influence the modeled comparative effectiveness of PGT-A. This was reflected in the sensitivity analysis for women aged 41–42 years (Fig. 6B). Nevertheless, the PGT-A test cost was the most influential parameter in this age group. These findings underscore the importance of considering variability in euploidy rates when interpreting the generalizability of the model across different laboratories and PGT-A platforms. Nevertheless, a major strength of the model is its flexibility, which allows the incorporation of newly published, relevant data on IVF in Israel as well as data from other subpopulations and different PGT-A platforms.

Our model demonstrated that live birth probabilities of PGT-A was consistently inferior to that of the no-PGT-A approach. The lower effectiveness observed with PGT-A should be interpreted in the context of the model assumptions rather than as evidence that PGT-A itself adversely affects live birth. The model assumes no detrimental effect of embryo biopsy on implantation; therefore, the difference between the strategies arises primarily from embryo classification and selection. In the PGT-A arm, only embryos classified as euploid were considered eligible for transfer, whereas in the morphology-based strategy all usable blastocysts remained available for sequential transfer. Consequently, exclusion of potentially viable embryos following PGT-A classification may reduce the cumulative pool of embryos available for transfer and, thereby, the modeled probability of achieving a live birth. This may occur because of false-positive classification or biological complexities such as embryonic mosaicism. Importantly, our model did not allow transfer of embryos classified as mosaic, although mosaic embryos (with low-level mosaicism), and occasionally embryos with segmental aneuploid findings, may be considered for transfer in clinical practice. Thus, the model may underestimate the reproductive potential of the PGT-A strategy by excluding embryos that could potentially result in a live birth. Accordingly, the lower live birth probabilities associated with PGT-A should be regarded as conditional on the classification and transfer assumptions incorporated into the model and not as an inherent adverse effect of PGT-A.

Although the clinical benefits of PGT-A continue to be a subject of debate, its implementation is associated with a reduced miscarriage rate per pregnancy and per transfer. Besides the potential economic implications, PGT-A serves as a valuable tool for mitigating the emotional distress caused by miscarriages and unsuccessful embryo transfers in certain patients. Moreover, beyond the emotional difficulties, there are populations, such as ultra-Orthodox, for whom termination of pregnancy is religiously prohibited even in cases of chromosomally abnormal fetuses. These factors could affect patient preferences and potentially influence public funding in certain adverse clinical situations.

### In conclusion

From a payer perspective, PGT-A should not be universally funded in the Israeli public health system, which provides extensive IVF coverage. Instead, selective and conditional coverage for advanced age women may be considered only if test costs are reduced. Future research should aim to incorporate real-world data from Israeli clinics, develop models that account for effectiveness per oocyte retrieval, and explore the cost-effectiveness of newer technologies, such as AI-driven embryo selection. As technology advances and test costs decrease, periodic reevaluation of the economic landscape will become increasingly important.

## Data Availability

Data sharing is not applicable to this article as no new datasets were generated during the current study. All model inputs were derived from previously published studies and sources cited in the manuscript.

## Abbreviations

ART: Assisted Reproductive Technologies
BHCG: Beta Human Chorionic Gonadotropin
HFEA: Human Fertilization and Embryology Authority
ICER: Incremental Cost-Effectiveness Ratio
IVF: In Vitro Fertilization
MoH: Ministry of Health
NGS: Next Generation Sequencing
NIS: New Israeli Shekels
OHSS: Ovarian Hyper-Stimulation Syndrome
OPU: Ovum Pick Up
PGT: A-Preimplantation Genetic Testing for Aneuploidy
RIF: Repeated Implantation Failure
SART: Society for Assisted Reproduction Technology
SART CORS: Society for Assisted Reproductive Technology Clinic Outcome Reporting System
SET: Single Embryo Transfer
U.S.: United States
